# Irisin, a promising adipomyokine, shows low levels in relation to periodontal diseases, unlike visfatin and IL-6 (case-control study)

**DOI:** 10.3389/froh.2025.1614433

**Published:** 2025-11-06

**Authors:** Farah Sabah Salim, Saif S. Saliem

**Affiliations:** Department of Periodontics, College of Dentistry, University of Baghdad, Baghdad, Iraq

**Keywords:** IL-6, irisin, visfatin, periodontitis, gingivitis, saliva

## Abstract

**Introduction:**

Adipose tissue releases pro- and anti-inflammatory cytokines and hormones such as irisin, visfatin, and interleukin-6, which may be linked to periodontal diseases.

**Objectives:**

Our study aimed to determine salivary irisin, visfatin, and interleukin-6 levels in gingivitis and periodontitis patients, compare them with healthy periodontal patients, and evaluate the association between these biomarkers.

**Materials and methods:**

Ninety participants were involved in this case-control study: 30 patients diagnosed with periodontitis (P), 30 patients with gingivitis (G), and 30 periodontally healthy subjects (control; C). The periodontal clinical parameters were documented. The ELISA test examined irisin, visfatin, and interleukin-6 saliva levels. Data were analyzed using SPSS (V.29).

**Results:**

Irisin significantly decreased in gingivitis and periodontitis compared to the control group (*p* < 0.05). In contrast, gingivitis and periodontitis reported elevated levels of visfatin and interleukin-6 compared to the control group (*p* < 0.05). Visfatin levels did not significantly change between gingivitis and periodontitis (*p* > 0.05). All periodontal clinical measures showed a significant negative association with irisin (*p* < 0.05), except plaque index in the gingivitis group. At the same time, visfatin and interleukin-6 showed significant positive relationships with all clinical periodontal markers (*p* < 0.05).

**Conclusions:**

Salivary irisin levels were reduced in individuals with gingivitis and periodontitis, whereas visfatin and interleukin-6 levels were elevated. These biomarkers may predict susceptibility to periodontal disease.

## 1 Introduction

Periodontal disease (PD) is an inflammatory condition closely linked to bacterial infections (1, 2); it develops through complex interactions between harmful bacteria below the gum line and the body's immune response (3). The development and severity of PD are significantly influenced by genetic susceptibility, as well as environmental factors such as smoking and systemic disorders (4). Two predominant forms of PD are gingivitis and periodontitis. The search for the specific bacteria that cause periodontal disease is ongoing; however, recent findings indicate that the body's inflammatory response plays a crucial role in affecting the bacteria in the gums, thereby accelerating the onset of periodontitis (5). Adipose tissue, which was once considered merely a fat storage site, actually secretes various bioactive substances known as adipokines (6), such as interleukin-6 (IL-6), visfatin, and irisin, that can influence inflammatory processes (7). Irisin, a newly identified adipomyokine, mediates the effects of physical exercise, with primary production occurring in adipose tissue and skeletal muscle (8). It regulates metabolic processes and helps maintain the balance between bone growth and resorption (9). Irisin has anti-inflammatory effects and may contribute to metabolic regulation (10). In the context of periodontitis, irisin may help modulate the inflammatory response and potentially protect against periodontal tissue destruction (11). Visfatin, another adipokine primarily derived from adipose tissue (12), but also produced in other tissues including periodontal tissue (13), Visfatin is generally expression elevated in inflammatory conditions (14), This leads to an increase in both pro-inflammatory and anti-inflammatory cytokines, such as interleukin-1 beta (IL-1β) and interleukin-6 (IL-6) (15). IL-6 is produced by various cell types, including adipocytes, macrophages, fibroblasts, endothelial cells, and skeletal muscle, during the early phases of inflammation (16). It serves as a significant mediator in inflammatory processes and regulates acute-phase proteins (17). Moreover, IL-6 plays a vital role in the progression of periodontal disease by promoting the formation of cells responsible for bone breakdown, which increases bone loss while decreasing bone growth (18). Elevated visfatin levels can lead to increased IL-6 release (15), creating a cycle of inflammation that exacerbates periodontal disease. In this context, irisin may disrupt this cycle, potentially lowering IL-6 levels and reducing inflammation (10). To the best of our knowledge, no research has yet investigated the role of irisin in patients with plaque-induced gingivitis. Therefore, this study will be the first to explore the significance of irisin in this context. Additionally, while there are two studies addressing irisin in the case of periodontitis (8, 19), none have examined its relationship with visfatin and interleukin-6 in the etiology of periodontal disease (PD). By understanding these connections, we aim to shed light on the inflammatory mechanisms involved in PD and to identify potential therapeutic targets for intervention.

## 2 Materials and methods

### 2.1 Study design

Ninety systemically healthy subjects (30 with healthy periodontium, 30 with generalized gingivitis, and 30 with generalized periodontitis) participated in this case-control study. The individuals selected for the study were patients who visited the College of Dentistry/University of Baghdad. Clinical periodontal information, including plaque index (PI), bleeding on probing (BOP), probing pocket depth (PD), and clinical attachment loss (CAL), is documented in periodontal records for every patient. A calibrated examiner conducted a complete mouth examination with a periodontal probe (UNC 15), assessing six locations per tooth, excluding plaque scores, and utilising a disclosing agent for four surfaces. Body mass index (BMI) would be <30 and determined by dividing the weight of the body in kilograms (kg) by height squared in meters (m) (20), and the waist-to-height ratio would be <0.5 and determined by dividing the waist circumference by height (21). The subjects were divided into:

The control group consisted of periodontally healthy individuals. Eligible healthy participants had no indications of inflammation, fewer than 10% of periodontal sites exhibiting bleeding on probing with no probing depth ≥3 mm at any sites, and no signs of radiographic bone loss (22).

Generalized gingivitis (G), dental biofilm-induced gingivitis, is characterized by the presence of bleeding sites ≥30% with probing depths ≤3 mm, and lack of CAL (22).

Generalized periodontitis (P). Characterized as interdental CAL identifiable at more than two nonadjacent teeth accompanied by probing depths exceeding 3 mm, it is observable at two or more teeth; all periodontitis cases must exhibit generalized periodontitis (≥30% of teeth involved) and unstable status (PPD ≥5 mm or PPD 4 mm with BOP), then conducted according to stages. (Stage I: interdental CAL at 1–2 mm, stage II: interdental CAL 3–4 mm, stage III and stage IV: interdental CAL was ≥5 mm (23).

### 2.2 Inclusion criteria

Have a minimum of 20 teeth, be systemically healthy patients, not be under medication for the last three months, and have a waist-to-height ratio (WHtR) <0.5 and a BMI <30.

### 2.3 The exclusion criteria

Pregnant or lactating mothers or users of contraceptive medications; patients who used antibiotic therapy, anti-inflammatory treatment, or immunosuppressive medicine in the preceding three months; smokers or alcoholics taking medications that may induce adverse complications, such as gingival enlargement; individuals who underwent any periodontal interventions within the past three months before registration; patients receiving orthodontic treatment or dental implants; and those with oral conditions not associated with periodontitis, such as aphthous ulcers or lichen planus are all excluded.

### 2.4 Sample size determination

The analysis power performed using G*Power software established the sample size of this study. At an alpha level of 0.05, a power of 0.95, and an anticipated modest effect size, a test determined a minimal sample size of 69 individuals for all groups. To accommodate probable attrition and enhance statistical power, we enlisted 30 volunteers in each group, yielding a total sample size of 90 participants.

### 2.5 Sample collection and laboratory analysis

Saliva samples were collected from all patients without stimulation. Participants abstained from eating, drinking, or using any oral hygiene products for at least two hours before specimen collection, which took place between 9:00 and 11:00 A.M. To minimize the immediate effects of physical exertion, participants were asked to remain seated and relaxed for at least 1 h before the sample collection. Samples were obtained before the clinical periodontal assessment. After being centrifuged at 10,000 rpm for 10 min, all saliva samples were transferred into Eppendorf tubes and stored at −20°C until the analysis day (24). To measure irisin, visfatin, and IL-6 levels, we utilized specialized ELISA kits (Human ELISA Kit, Elabscience) according to the manufacturer's instructions. Salivary biomarkers were quantified using commercially available ELISA kits (Elabscience®, Houston, TX, USA) according to the manufacturer's instructions: Human Irisin (Catalog No: E-EL-H6120; detection range: 15.63–1,000 pg/ml; sensitivity: 9.38 pg/ml), Human Interleukin-6 (IL-6) (Catalog No: E-EL-H0102c; detection range: 7.81–500 pg/ml; sensitivity: 4.69 pg/ml), and Human Visfatin (Catalog No: E-EL-H1330c; detection range: 0.313–20 ng/ml; sensitivity: 0.19 ng/ml). All kits demonstrate high specificity for the target analyte without significant cross-reactivity with related analogues.

### 2.6 Statistical analyses

All analyses used commercial statistical software packages, including SPSS (version 29, IBM, USA) and GraphPad Prism (version 9). The Shapiro–Wilk test assessed the data distribution, which indicated a normal distribution. Consequently, multi-group comparisons were performed using the ANOVA test, and further intra-group comparisons were conducted with the Dunnett *post hoc* tests and the Bonferroni test. The chi-square test and its adjustments were utilized to analyze qualitative data. Additionally, Pearson correlation analysis was employed to examine the relationships among parameters that followed a normal distribution. Each statistical test had a significance level set at *α* = 0.05. In addition to the primary analyses, we performed multivariate regression models adjusting for potential confounders available in our dataset, including age, body mass index (BMI), waist-to-height ratio (WHtR), and number of teeth to evaluate the independent association between periodontal diseases and biomarkers levels.

## 3 Results

### 3.1 Clinical findings

When compared to individuals with gingivitis and healthy controls, clinical periodontal parameters, specifically plaque index (PI), bleeding on probing (BOP), probing depth (PD), and clinical attachment level (CAL), were significantly higher in the periodontitis group (*p* = 0.000, see Table 1). Furthermore, patients with gingivitis exhibited elevated PI and BOP levels compared to those with healthy periodontal conditions (see Table 1). Body mass index (BMI) and waist-to-height ratio (WHtR) did not show statistically significant differences among the periodontitis, gingivitis, and control groups for either measure (*p* = 0.050 and *p* = 0.190, respectively; see Table 1). Stages III and IV also displayed significantly higher clinical periodontal parameters than stages I and II (see Table 2).

**Table 1:** Demographic, clinical, and biochemical parameters of control groups, gingivitis, and periodontitis.

Parameters	Control (C) *n* = 30	Gingivitis (G) *n* = 30	Periodontitis (P) *n* = 30	*p*-value
**Age (years)	28.03 ± 8.373	26.23 ± 7.104	38.67 ± 13.299	0.001
*Gender F/M	15/15	15/15	15/15	1.00
**BMI (kg/m^2^)	23.49 ± 2.98	23.40 ± 3.32	25.22 ± 3.38	0.050
**WHtR	0.47 ± 0.03	0.45 ± 0.03	0.47 ± 0.04	0.190
**PI (%)	8.12 ± 2.99	63.66 ± 19.369	70.96 ± 13.12	0.000
**BOP (%)	6.54 ± 2.23	52.76 ± 15.08	68.36 ± 12.48	0.000
*PPD (mm)	1.12 ± 0.14	1.69 ± 0.23	4.57 ± 0.41	0.000
*CAL (mm)	–	–	4.70 ± 1.46	0.000
**Irisin (pg/ml)	275.10 ± 32.98	176.24 ± 68.68	140.82 ± 20.24	0.000
**Visfatin (pg/ml)	2.27 ± 0.62	4.67 ± 1.71	5.25 ± 0.66	0.000
**IL-6 (pg/ml)	10.82 ± 4.55	23.67 ± 7.44	28.11 ± 1.79	0.000

BMI, body mass index; WHtR, waist-to-height ratio; PI, plaque index; PPD, probing pocket depth; BOP, bleeding on probing; CAL, clinical attachment lost; IL-6, interleukin-6, data shown as mean ± standard deviation.

* Student *t* test.

** ANOVA Statistical difference with the control group *p* < 0.05.

**Table 2:** Demographic, clinical, and biochemical parameters among periodontitis group (stage I + II and stage III + IV).

Variables	Stage I + II *n* = 11	Stage III + IV *n* = 19	*P* value
**Irisin (pg/ml)	148.75 ± 20.65	136.23 ± 19.06	0.127
**Visfatin (pg/ml)	4.9 ± 20.70	5.4 ± 50.56	0.045
**IL-6 (pg/ml)	26.94 ± 1.18	28.79 ± 1.74	0. 003
**PI%	60.55 ± 13.30	76.99 ± 8.67	0.003
**BOP%	63.44 ± 11.50	71.21 ± 12.42	0.149
*PPD(mm)	4.51 ± 0.21	5.39 ± 0.46	0.000
*CAL(mm)	3.55 ± 0.84	5.71 ± 1.23	0.000

Data shown as mean ± standard deviation.

* Comparison done by independent *t*-test.

** Comparison done by Dunnett's test. Significant difference at *p* < 0.05.

### 3.2 Biochemical findings

**Figure 1:**
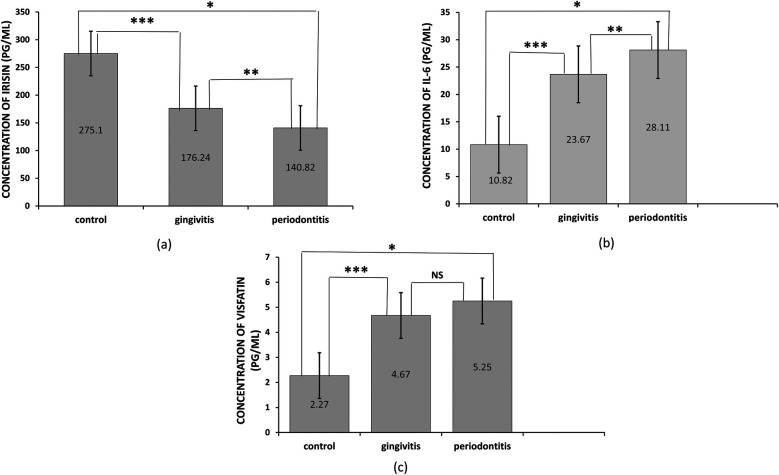
Levels of salivary irisin, visfatin, and interleukin-6 (IL-6) in different groups. **(a)** Irisin concentration, the asterisk symbol (*) indicates a significant difference between periodontitis and control at *p* < 0.000, (**) indicates a significant difference between periodontitis and gingivitis at *p* = 0.036, (***) indicates a significant difference between gingivitis and control at *p* = 0.001, **(b)** IL-6 concentration, the asterisk symbol (*) indicates a significant difference between periodontitis and control at *p* < 0.000, (***) indicates a significant difference between gingivitis and control at *p* < 0.000, **(c)** Visfatin concentration, the asterisk symbol (*) indicates a significant difference between periodontitis and control at *p* < 0.000, while there was no significant difference (NS) between gingivitis and periodontitis. Intergroup differences were determined using one-way ANOVA followed by a Bonferroni *post hoc* test for multiple comparisons. Bars represent the mean, while error bars represent the standard deviation.

The concentration levels of irisin, visfatin, and interleukin-6 (IL-6) are presented in Table 1. Patients with periodontitis had lower salivary irisin levels compared to individuals with gingivitis and healthy controls (*p* = 0.036 and *p* = 0.000, respectively; see Figure 1). Moreover, irisin levels were also decreased in the gingivitis group compared to the control group (*p* = 0.001; see Figure 1). In contrast, visfatin and IL-6 levels were significantly elevated in the periodontitis group when compared to both the gingivitis and control groups (*p* = 0.000 for both; see Figure 1). In patients with gingivitis, visfatin and IL-6 levels were higher than in the control group (*p* = 0.000 for both). It is noteworthy that there was no significant difference in visfatin levels between the gingivitis and periodontitis groups (*p* = 0.06; see Figure 1). Furthermore, visfatin and IL-6 demonstrated high statistical significance in stages I and II compared to stages III and IV. In contrast, no statistical differences in irisin levels were found between stages I and II and stages III and IV (see Table 2).

### 3.3 Correlations

**Figure 2:**
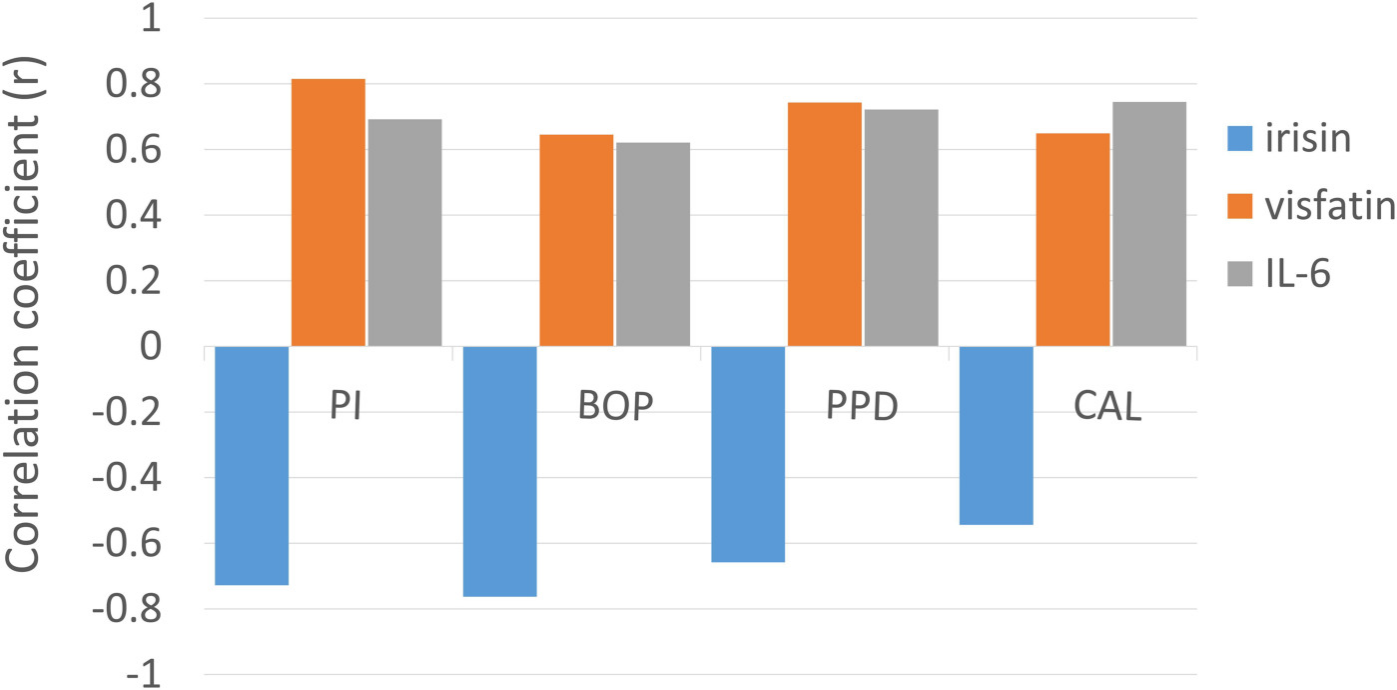
Correlation between periodontal parameters (PI, BOP, PPD, and CAL) in the periodontitis group with salivary biomarkers (irisin, visfatin, and IL-6). The height of the bars indicates the strength of the correlation, while the polarity (positive or negative) reflects the direction of the relationship, as determined by Pearson correlation coefficients (*r*).

In the gingivitis group, irisin demonstrated a moderate, statistically significant negative correlation with bleeding on probing (BOP) (*p* < 0.05, Table 3). In contrast, both visfatin and interleukin-6 (IL-6) showed moderate, statistically significant positive relationships with plaque index (PI) and BOP (*p* < 0.05, Table 3). Within the periodontitis group, irisin exhibited a significant negative correlation with all clinical periodontal measures, including PI, BOP, probing pocket depth (PPD), and clinical attachment loss (CAL) (*p* < 0.05, Table 3; Figure 2). Conversely, visfatin and IL-6 were positively associated with all clinical parameters of periodontal disease (*p* < 0.05, Table 3; Figure 2), with a particularly strong positive correlation between PI and visfatin (r = 0.815, *p* < 0.001, Table 3). Moreover, a moderate negative correlation between irisin and visfatin was observed in both the gingivitis and periodontitis groups (Table 3) and within the stage I + II and stage III + IV subgroups (Table 4). A negative association between irisin and IL-6 was identified across all examined groups (Tables 3, 4). In contrast, visfatin and IL-6 maintained a consistent and significant positive correlation across all groups, with the strongest relationship found in the stage I + II subgroup (*r* = 0.640, *p* = 0.033; Table 4). After adjusting for age, BMI, WHtR and number of teeth in multivariate analyses, the association between periodontitis and irisin levels remained significant (*β* = −0.232, *p* = 0.001, Table 5). These findings indicate that the observed relationship is not explained solely by these confounding factors.

**Table 3:** Correlations between biomarkers and periodontal clinical parameters (Pearson correlation coefficients, *r* values).

Variables		Irisin		Visfatin		IL-6	
		*r*	*p*-value	r	*p*-value	*r*	*p*-value
Control	Irisin (pg/ml)						
	visfatin (pg/ml)	0.173	0.359				
	IL-6 (pg/m)	−0.251	0.180	0.714	*0.000		
	PLI%	0.170	0.367	0.223	0.235	0.110	0.559
	BOP%	0.112	0.553	0.155	0.411	0.049	0.795
	Age (year)	−0.036	0.847	−0.200	0.289	−0.179	0.342
	BMI (kg/m^2^)	−0.008	0.969	−0.098	0.608	−0.160	0.397
	WHtR	−0.189	0.316	0.139	0.461	0.177	0.348
Gingivitis	Irisin (pg/ml)						
	visfatin(pg/ml)	−0.394	*0.031				
	IL-6 (pg/ml)	−0.395	0.051	0.503	*0.004		
	PLI%	0.137	0.467	0.518	*0.003	0.457	*0.011
	BOP%	−0.412	*0.023	0.495	*0.005	0.461	*0.010
	Age (year)	−0.065	0.730	0.253	0.176	0.176	0.350
	BMI (kg/m^2^)	−0.160	0.390	0.223	0.234	−0.010	0.957
	WHtR	−0.050	0.724	0.017	0.925	−0.191	0.311
Periodontitis	Irisin (pg/ml)						
	visfatin (pg/ml)	−0.701	*0.000				
	IL6 (pg/ml)	−0.446	*0.013	0.618	*0.000		
	PLI%	−0.728	*0.000	0.815	*0.000	0.692	*0.000
	BOP%	−0.763	*0.000	0.645	*0.000	0.621	*0.000
	PPD (mm)	−0.658	*0.000	0.743	*0.000	0.722	*0.000
	CAL (mm)	−0.544	*0.000	0.649	*0.000	0.745	*0.001
	Age (year)	−0.105	0.578	0.237	0.206	0.027	0.884
	BMI (kg/m^2^)	0.125	0.510	0.042	0.823	−0.144	0.445
	WHtR	−0.057	0.761	0.029	0.877	−0.216	0.251

Pearson correlation test.

* *p* < 0.05.

**Table 4:** Correlations between biomarkers and demographic data within the periodontitis group (stage I + II, stage III + IV).

Variables		Irisin		Visfatin		IL-6	
		*r*	*p*-value	*r*	*p*-value	*r*	*p*-value
Stage I + II	Irisin (pg/ml)						
	Visfatin (pg/ml)	−0.705	0.015				
	IL-6 (pg/ml)	−0.118	0.727	−0.635	0.033		
	PLI%	−0.636	0.035	0.868	0.000	0.638	0.034
	BOP%	−0.623	0.040	0.699	0.016	0.684	0.020
	PPD (mm)	−0.625	0.039	0.781	0.004	0.672	0.023
	CAL (mm)	−0.614	0.044	0.763	0.006	0.674	0.022
Stage III + IV	Irisin (pg/ml)						
	visfatin (pg/ml)	−0.635	0.003				
	IL-6 (pg/ml)	−0.465	0.044	0.546	0.015		
	PLI%	−0.821	0.000	0.717	0.000	0.568	0.008 S
	BOP%	−0.727	0.000	0.604	0.006	0.534	0.018 S
	PPD (mm)	−0.679	0.001	0.654	0.002	0.595	0.007 S
	CAL (mm)	−0.486	0.034	0.486	0.034	0.707	0.000 S

(Pearson correlation coefficients (*r* values).

**Table 5:** Multiple linear regression models for salivary biomarker levels.

Model I	Visfatin (Pg\ml)			IL-6 (Pg\ml)			Irisin (Pg\ml)		
	*(R^2^ = 0.771)*			*(R^2^ = 0.786)*			*(R^2^ = 0.740)*		
	*B*	*SE*	*P*	*B*	*SE*	*P*	*B*	*SE*	*P*
Il-6	*0* *.* *046*	*0* *.* *076*	*0* *.* *550*	*0* *.* *391*	*0* *.* *643*	*0* *.* *550*	*−10* *.* *054*	*6* *.* *928*	*0* *.* *162*
Irisin	*−0* *.* *009*	*0* *.* *007*	*0* *.* *162*	*−0* *.* *035*	*0* *.* *018*	*0* *.* *067*	*4* *.* *464*	*2* *.* *302*	*0* *.* *062*
No. of teeth	*−0* *.* *060*	*0* *.* *029*	*0* *.* *048*	*0* *.* *049*	*0* *.* *091*	*0* *.* *593*	*−1* *.* *371*	*0* *.* *981*	*0* *.* *178*
BMI	*0* *.* *027*	*0* *.* *025*	*0* *.* *299*	*−0* *.* *059*	*0* *.* *075*	*0* *.* *435*	*1* *.* *183*	*0* *.* *808*	*0* *.* *159*
WHtR	*−1* *.* *217*	*2* *.* *379*	*0* *.* *607*	*−11* *.* *283*	*6* *.* *323*	*0* *.* *090*	*−47.670*	*75* *.* *624*	*0* *.* *536*
Age	*0* *.* *434*	*0* *.* *564*	*0* *.* *065*	*0* *.* *547*	*0* *.* *342*	*0* *.* *232*	*0* *.* *341*	*0* *.* *386*	*0* *.* *655*
PL%	*0* *.* *028*	*0* *.* *012*	*0* *.* *035*	*0* *.* *010*	*0* *.* *040*	*0* *.* *809*	*−0.297*	*0* *.* *443*	*0* *.* *510*
BOP %	*1* *.* *001*	*0* *.* *010*	*0* *.* *040*	*0* *.* *047*	*0* *.* *027*	*0* *.* *009*	*−0* *.* *650*	*0* *.* *290*	*0* *.* *036*
PPD (ml)	*−0* *.* *152*	*0* *.* *245*	*0* *.* *542*	*1* *.* *429*	*0* *.* *633*	*0* *.* *038*	*−16* *.* *156*	*7* *.* *199*	*0* *.* *036*
CAL (ml)	*0* *.* *049*	*0* *.* *117*	*0* *.* *052*	*0* *.* *161*	*0* *.* *339*	*0* *.* *040*	*−4* *.* *059*	*3* *.* *718*	*0* *.* *048*
Gingivitis	*0* *.* *412*	*0* *.* *114*	*0* *.* *034*	*0* *.* *012*	*0* *.* *898*	*0* *.* *004*	*−0* *.* *310*	*0* *.* *897*	*0* *.* *054*
Periodontitis	*0* *.* *034*	*0* *.* *898*	*0* *.* *000*	*0* *.* *565*	*0* *.* *989*	*0* *.* *074*	*−0* *.* *845*	*0* *.* *908*	*0* *.* *000*
Model II	*(R^2^ = 0.595)*			*(R^2^ = 0.678)*			*(R^2^ 0.700)*		
No. of teeth	*−0* *.* *039*	*0* *.* *002*	*0* *.* *039*	*–*	*–*	*–*	*–*	*–*	*–*
PL%	*0* *.* *021*	*0* *.* *009*	*0* *.* *002*	*–*	*–*	*–*	*–*	*–*	*–*
BOP%	*0* *.* *031*	*0* *.* *786*	*0* *.* *023*	*1* *.* *122*	*0* *.* *988*	*0* *.* *022*	*−0.897*	*0* *.* *239*	*0* *.* *004*
PPD (ml)	*–*	*–*	*–*	*−1* *.* *234*	*0* *.* *034*	*0* *.* *001*	*−0* *.* *127*	*0* *.* *33*	*0* *.* *001*
CAL (ml)	*–*	*–*	*–*	*−1* *.* *343*	*0* *.* *234*	*0* *.* *002*	*−3.676*	*2* *.* *823*	*0* *.* *003*
Gingivitis	*0* *.* *897*	*0* *.* *112*	*0* *.* *002*	*0* *.* *123*	*0* *.* *232*	*0* *.* *003*	*–*	*–*	*–*
Periodontitis	*0* *.* *45*	*0* *.* *675*	*0* *.* *000*	*–*	*–*	*–*	*−0.232*	*0* *.* *786*	*0* *.* *001*

### 3.4 Diagnostic potential of the biomarkers

**Figure 3:**
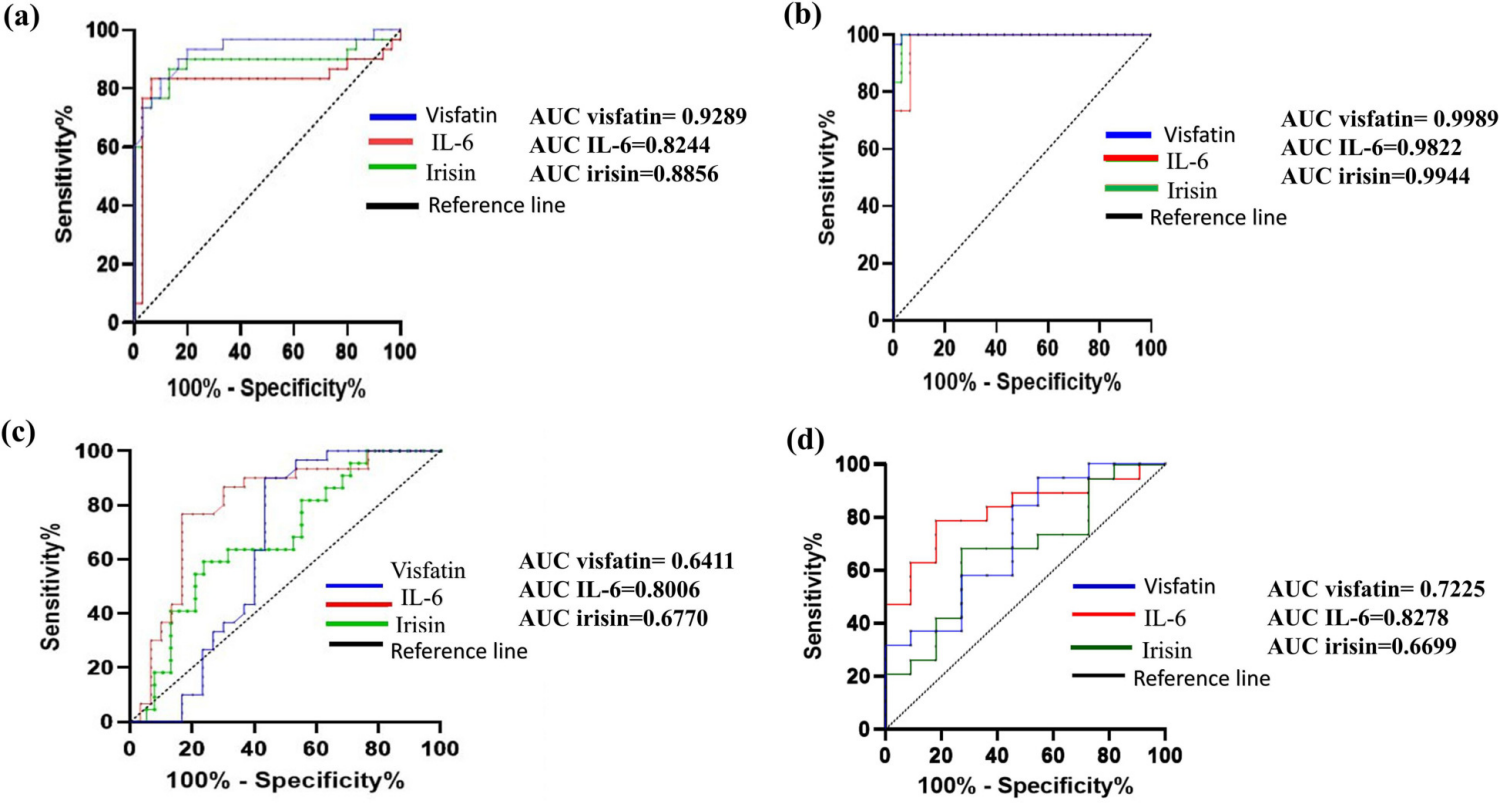
Receiver operating characteristic (ROC) curves demonstrating the diagnostic performance of irisin, visfatin, and IL-6 in various comparisons: **(a)** control Vs gingivitis, **(b)** control vs. periodontitis, **(c)** gingivitis vs. periodontitis, **(d)** stage I + II vs. stage III + IV. Each curve illustrates the sensitivity and specificity of biomarkers, with the area under the curve (AUC) indicating predictive accuracy. The AUC values for each biomarker are displayed in each panel, with higher AUC values signifying higher diagnostic accuracy.

The diagnostic efficacy of irisin, visfatin, and interleukin-6 (IL-6) biomarkers was evaluated using receiver operating characteristic (ROC) curve analysis (Figure 3). All three biomarkers effectively differentiated healthy individuals from those with periodontitis, with area under the curve (AUC) values of 0.9944% for irisin, 0.9989% for visfatin, and 0.9822% for IL-6. They also did well at distinguishing healthy controls from gingivitis patients. IL-6 showed satisfactory diagnostic performance in separating gingivitis patients from those with periodontitis and identifying early (Stage I + II) and advanced (Stage III + IV) stages of periodontitis. Conversely, neither irisin nor visfatin demonstrated adequate diagnostic precision to differentiate between gingivitis and periodontitis or among the different stages of periodontal disease.

## 4 Discussion

Inflammatory response to periodontal diseases is a crucial survival mechanism (25). Chronic inflammatory disorders are linked with the activation of detrimental signal transduction pathways and the release of many inflammatory mediators, all of which play their roles in disease development. This study demonstrated significantly higher salivary concentrations of visfatin and interleukin-6 (IL-6) in patients with gingivitis and periodontitis relative to periodontally healthy controls. Irisin concentrations were significantly reduced in the gingivitis and periodontitis groups. Notably, this study is the first to show a link between lower levels of salivary irisin and periodontal inflammation, suggesting that irisin might negatively affect the inflammation processes involved in these periodontal diseases. Irisin, a myokine identified in 2012 and induced by physical exercise, is crucial in regulating glucose and lipid homeostasis (26). On the other hand, pro-inflammatory molecules such as visfatin and interleukin-6 (IL-6) are known to contribute to insulin resistance, unbalanced fat metabolism, oxidative stress, and systemic inflammation (27, 28). Irisin has been shown to provide protective effects by enhancing insulin sensitivity, maintaining metabolic equilibrium, and reducing oxidative stress (29). The findings highlight the inverse relationship observed in our study between irisin levels and those of visfatin and IL-6.

This study employed a case-control design because it offers specific advantages over other study designs. Case-control studies are comparatively dependable and economical. Notably, the salivary gland, serous, and mucous acinar cells are reported to generate irisin (30). Therefore, measuring irisin levels in saliva may provide a more accurate insight into the role of this protein in periodontal diseases. However, to date, no study has evaluated irisin's probable role in the development of plaque-induced gingivitis patients or the pathogenesis of irisin together with visfatin and IL-6 in periodontitis.

During physical activity, peroxisome proliferator-activated receptor gamma coactivator 1-alpha (PGC-1α) in skeletal muscle releases fibronectin type III domain-containing protein 5 (FNDC5). Once FNDC5 is released, it undergoes cleavage by an unknown protease, creating irisin (31). Since its discovery, irisin has received much interest due to its proposed critical role in several biological processes, including inflammation. Reports suggest that irisin may have differential effects on NF-κB, depending on the biochemical pathways involved (10). In context of inflammation, irisin might reduce the activity of NF-κB via a reduction in toll-like receptor 4/nuclear factor erythroid 2–related factor (TLR4/NRF2) and AMPK signaling pathways. This could lead to lower release of inflammatory markers such as COX-2, IL-1β, iNOS, TNF-α, and IL-6 (32). It is plausible that decreased irisin levels in individuals with inflammatory conditions could contribute to heightened inflammatory responses associated with impaired NF-κB regulation. This may help to explain our observation of a negative correlation between irisin and IL-6. In contrast, visfatin, produced in human gingival fibroblasts (hGFs) and human periodontal ligament cells (hPDLCs), is thought to regulate pro-inflammatory and pro-degradative factors partially through the NF-*κ*B signaling pathway, thus promoting an increased expression of IL-6 and increased inflammation (15). This is consistent with our finding of a positive correlation between visfatin and IL-6. Notably, this activation of NF-κB may suppress irisin production, while irisin could counteract their effect by inhibiting NF-κB activity. We propose that irisin may play a pivotal role in regulating the balance between adipokines such as visfatin and IL-6 within the context of periodontal disease. It is hypothesized that an increase in irisin levels could potentially mitigate the inflammatory effects of visfatin and IL-6, suggesting a possible protective mechanism against the systemic inflammation often associated with periodontal disease. Supporting this notion, a study by Yuksel Ozgor et al. demonstrated that the mice injected with irisin showed decreased pro-inflammatory adipokines, weight gain, and lower visfatin levels (33). This aligns with our findings of a negative correlation between irisin and visfatin. Moreover, Khajebishak et al. found that irisin levels in obese people with type 2 diabetes were considerably lower than in controls, and a negative correlation between irisin and IL-6 was observed (34), reinforcing our observation of a negative correlation between irisin and IL-6. Furthermore, research on metabolic disorders, including Gestational Diabetes Mellitus (35, 36), has suggested that reduced irisin levels may be linked to obesity and diabetes, which are implicated in the etiology of periodontitis (37). While several studies have shown reduced irisin levels, such as myocardial damage associated with severe hypothyroidism (38), cardiovascular diseases (39), and rheumatoid arthritis (40), which align with our findings, other research has indicated elevated irisin levels (8) as a comenstary mechanisim to inflammation however despite variations in study outcomes, a prevailing hypothesis reduced irisin levels in persons with inflammatory metabolic disorders.

For the first time, the study identifies a negative correlation between irisin and all periodontal clinical parameters, alongside positive correlations between clinical periodontal parameters and saliva visfatin and IL-6 levels, which agrees with prior research (41). Irisin levels are known to rise with increased visceral adiposity (42); however, our subjects exhibited a waist-to-height ratio (WHtR) below 0.5, a metric regarded as a more precise predictor of visceral fat distribution than body mass index (BMI) (43). Our findings support the use of WHtR as a more effective anthropometric measure than BMI, which, despite its widespread use, is often critiqued for inadequately representing adiposity distribution (44). Interestingly, our study found no statistically significant correlation between the examined biomarkers and BMI or WHtR. These findings align with specific prior publications (2, 45), while they diverge from others (8). Therefore, more research with bigger and more varied groups is warranted to elucidate the potential link between these biomarkers and anthropometric measures such as BMI and WHtR. One limitation of this study is the lack of quantitative data on participants' physical activity. Our results may be confounded by irisin, a myokine whose circulating concentration is affected by acute and chronic physical activity. Although all participants were instructed to remain seated and relaxed for one hour prior to saliva sampling to minimize acute exercise-related fluctuations in irisin, we did not assess habitual levels of physical activity. Prior evidence shows that exercise can significantly alter baseline irisin concentrations: Boström et al. observed a 2–3 fold transient increase following acute exercise (46), while subsequent meta-analyses reported that chronic exercise interventions elevate circulating irisin by ∼20%–100% depending on exercise modality and intensity (47, 48). Thus, unmeasured variability in habitual physical activity may have biased our results, and future studies should objectively quantify physical activity to better disentangle its effects from the associations observed. Another limitation of this study is its small sample size. It is essential to acknowledge that, despite the irisin concentrations in this study being measured with a commercially available ELISA kit validated by the manufacturer for high specificity and absence of cross-reactivity with FNDC5 or other irisin analogues, the dependability of irisin quantification through ELISA continues to be a subject of ongoing discourse in the literature. The lack of a standardized assay and the variability among various commercial kits may affect the comparability of results across studies. This must be taken into account when interpreting the current results. An important consideration in interpreting our findings is the potential influence of confounding factors on circulating irisin levels. In the present study, we were able to adjust for age, BMI, and WHtR, and our results remained consistent after controlling for these variables, which strengthens the validity of our conclusions. However, other relevant factors such as detailed exercise habits, dietary intake, body fat percentage, and muscle mass were not available in our dataset. These variables may also contribute to variation in irisin secretion, and their absence represents a limitation of the current work. Therefore, our results should be interpreted with this limitation in mind, and we recommend that future research incorporate these additional measures to provide a more comprehensive understanding of the independent relationship between periodontal diseases and irisin levels.

## 5 Conclusion

With more precise testing methods in the future, the identification and detection of salivary irisin, visfatin, and IL-6 may serve as potential biomarkers to predict susceptibility to periodontal diseases.

## Data Availability

The datasets presented in this study can be found in online repositories. The names of the repository/repositories and accession number(s) can be found in the article/Supplementary Material.
